# Prevention of Clostridium Difficile Infection Among Hospitalized Elderly Patients Using Torani (Fermented Rice Water) and Xylitol Mixture Drink: The Study Protocol of an Open-Label Randomized Controlled Trial

**DOI:** 10.7759/cureus.85635

**Published:** 2025-06-09

**Authors:** Basanti Kumari Pathi, Jyoti Prakash Sahoo, Kumudini Panigrahi, Sidharth S Pattnaik, Santosh Kumar Dash, Shubhransu Patro, Debashish Mishra, Dipti Pattnaik, Manas Ranjan Behera, Manoja K Das

**Affiliations:** 1 Microbiology, Kalinga Institute of Medical Sciences, Bhubaneswar, IND; 2 Pharmacology, Kalinga Institute of Medical Sciences, Bhubaneswar, IND; 3 Internal Medicine, Kalinga Institute of Medical Sciences, Bhubaneswar, IND; 4 Neurology, Kalinga Institute of Medical Sciences, Bhubaneswar, IND; 5 Orthopaedics, Kalinga Institute of Medical Sciences, Bhubaneswar, IND; 6 Pediatrics, Kalinga Institute of Medical Sciences, Bhubaneswar, IND; 7 Public Health, The International Clinical Epidemiology Network (INCLEN) Trust International, New Delhi, IND

**Keywords:** clostridioides difficile infection, fermented diet, gut dysbiosis, gut-microbiota, microbial colonization, prebiotics, probiotics, short chain fatty acids, torani, xylitol

## Abstract

Background and objectives: *Clostridium difficile* infections (CDI) among elderly individuals are common. Antibiotic use and gut dysbiosis are major contributors to CDI. Probiotics and prebiotics help prevent CDI by addressing dysbiosis. This study aims to document the effect of the combination of probiotics from *Torani*, a traditional fermented food (rich in *Lactobacillus* species), and prebiotics (xylitol) on CDI among elderly hospitalized individuals.

Methods: This two-arm, open-label, randomized controlled trial (RCT) will be conducted at a tertiary care hospital in Odisha, India. The eligible elderly hospitalized participants will be randomized into two groups in a 1:1 ratio to receive 350 ml of either *Torani*-xylitol mixture or plain water once daily for 14 days. The data on sociodemography, clinical, and laboratory tests, antibiotics, and other medications used shall be recorded from their case sheets. Stool samples or rectal swabs will be collected on days 0 and 15 or at discharge/death/referral for CD isolation. A stool or rectal swab sample will be collected for any suspected CDI during the hospitalization. Follow-up contacts will be made on days 30 (+7), 60 (+7), and 90 (+7) to address any illness/infection, medication use, hospitalization, blood tests, and dietary practices.

Results: As we have not started the study, we do not have any observations yet.

Conclusions: This RCT shall document the effect of *the Torani*-xylitol mixture on CDI prevention and CD colonization among hospitalized elderly individuals. If found effective, it can promote using beneficial fermented foods to prevent CDI, reducing morbidity, mortality, hospitalization rate, and duration, cost of therapy, and bed occupancy.

## Introduction

*Clostridioides difficile* (also known as *Clostridium difficile*) infections (CDI) are linked to extended hospital stays and fatalities, particularly in the elderly population [1]. Though CDI is considered a nosocomial infection, community-based transmission among older adults is common [2]. An increase in CDI incidence has been observed in American, European, and certain Asian hospitals over time. This tendency can be attributed to the increasing antibiotic usage and the emergence of novel, hypervirulent strains [3-6]. In parallel with the rising CDI incidence, the antibiotic resistance pattern and associated mortality have also increased [7].

*Clostridioides difficile* (CD) usually spreads by the fecal-oral route and causes exotoxin-mediated illness. In India, CDI affects 3.4-18% of people, with older adults having a greater risk [8]. While 3-15% of healthy persons may have CD in their normal flora, 16-35% of hospitalized patients may have asymptomatic colonization [9,10]. The most significant risk factor for CDI is the use of antibiotics. The antibiotic class and its exposure duration determine the infection likelihood [11].

The resistance of the bacterial spores to heat and disinfectants contributes to the spread of CDI in hospitals. CDI ranges from mild diarrheal symptoms to fatal colitis [3,12]. Nowadays, community-acquired colitis is more prevalent owing to CDI and infective diarrhea [12]. Recurrent CDI (rCDI) increases morbidity, mortality, and healthcare costs and dampens the quality of life [3,12,13]. A recent meta-analysis shows 8.3 CDI cases for every 10,000 patient days [13]. Complex carbohydrate conversion by the normal colonic microbiota is essential to elicit resistance to invading microorganisms [14]. Gut microbiomes digest complex carbohydrates, starch, and dietary fiber to create short-chain fatty acids (SCFAs). SCFAs modulate inflammation and the immune system by lowering T and B cell differentiation and migrating immune cells (i.e., neutrophils, dendritic cells, and macrophages) [15].

Gut dysbiosis leads to acidification of the pH of the large intestine and, thereby, CD colonization and CDI [16]. Probiotics, prebiotics, and fecal microbiota transplantation have reduced CDI and CD colonization [17]. However, studies evaluating the additional benefits of SCFAs are scarce. Unlike probiotics and prebiotics, SCFAs are not easily available in the market. Xylitol, a transparent polyol, is a good substrate for SCFAs in the large intestine [18]. Various types of SCFAs, minerals, micronutrients, and commensal gut microbiota are abundant in fermented foods. Thus, they can positively impact immunomodulation, inflammation control, and gut microbiota regulation [19]. In India, many traditional fermented foods are consumed in households. These foods' cost, ease of administration, and sociocultural preparation render them good options for maintaining colonic ecology.

In the Indian state of Odisha, traditional fermented food includes Pakhala bhat (fermented rice) and *Torani* (fermented rice-water). In the *Torani*, the complex carbohydrates of rice are broken down by *Lactobacillus* species, namely *Lactobacillus plantarum*, *Lactobacillus casei*, and *Lactobacillus fermentum* [20,21]. Gas chromatography-mass spectrometry (GCMS) analysis reveals high SCFA concentration in the *Torani* [22]. In addition to altering phagocytosis and chemotaxis, the SCFAs have been demonstrated to produce reactive oxygen species (ROS) and alter cell proliferation. They also possess antibacterial, anti-inflammatory, and anticancer properties. *Torani*'s microbiota and SCFA concentration may reduce CD colonization and improve the gut microbiome [23,24].

Probiotics and prebiotics have been shown to lessen CD colonization [25,26]. The elderly are particularly at risk for CDI. Therefore, the primary focus for documentation on CD colonization and CDI prevention continues to be hospitals. This study aims to document the effect of a *Torani*-xylitol mixture (i.e., a combination of prebiotics, probiotics, and SCFAs) on the CDI and CD colonization status, along with the safety and tolerability of the elderly population.

## Materials and methods

Study design

This two-arm, open-label, randomized controlled trial (RCT) will be conducted at the Kalinga Institute of Medical Sciences (KIMS), Bhubaneswar, Odisha, India.

Participant and eligibility criteria

The patients aged above 60 years, of any gender, admitted to the hospital within the last 48 hours, anticipated to be admitted for at least seven days, and allowed orally or through the nasogastric tube will be included. The patients with known immunocompromised status (on high-dose steroids or immunosuppressants), scheduled for or who underwent any surgical procedure, on nil-per-oral (NPO) advice, admitted for only diagnostic tests or short-term assessment, requiring life support systems for unstable vitals, with renal failure or on fluid restriction, on any probiotics, with known food allergy or intolerance to xylitol, not complying with the study protocol, and very sick patients (death is thought to be imminent) will be excluded.

Study procedure

Investigational Product

The participants shall consume either of the two products under supervision. The products shall be prepared daily in the kitchen, packaged in similar bottles labeled with the participant ID, and supplied to the participants for daily consumption for up to 14 days.

Intervention Product

The participants in the intervention arm (Group A) shall receive the *Torani*-xylitol mixture containing 350 ml *Torani* (overnight fermented rice water prepared from cooked rice) and 5 gm xylitol once daily for up to 14 days. There is no prohibition on consuming the product with the meals.

Comparison Product

The participants in the comparator arm (Group B) shall be provided 350 ml of plain mineral water.

Randomization and Blinding

After obtaining informed consent, the participants (a total of 200 in the two groups) will be randomized into either of the two groups in a 1:1 ratio, following computerized permuted block randomization with block sizes of 4 and 6 (generated by an independent biostatistician using a web-based random number generator). Allocation concealment will be done using double-layered opaque envelopes. After obtaining consent from the participant, the research staff will inform the independent nurse (who will have the envelopes for randomization) to open the envelope and identify the group allocation, assign the study ID sequentially as per the envelope, and inform the study team member. Once opened for a participant, the envelope will not be used for another participant, even if the participant declines participation subsequently. The used envelopes shall be submitted to the independent statistician.

After opening the sealed envelope and allocating the participant, the study team member will inform the product preparation team (PPT) about the study ID and allocation group for the supply of the study product. The study products shall be prepared daily, packed in similar bottles, labeled with the participant’s ID (neither group nor product mentioned), and supplied individually to the concerned participant’s bedside. The products (i.e., *Torani*-xylitol mixture or plain mineral water) shall be supplied in sealed bottles labeled with the study IDs of the participants to the respective participant’s bedside daily (morning hours). The participants shall be asked to consume these drinks within two hours of supply, and the bottles shall be collected.

Due to the nature of the products, the participants and research staff cannot be blinded. The laboratory analysis and outcome-assessing teams will be blinded to the study group allocation. The bottles shall be selected to minimize the visualization of the product. For any reason, if required, the investigation product may be stopped (temporarily or completely) as per the instruction of the treating doctor.

Data Collection and Follow-Up

The data on sociodemography, anthropometry, clinical information (illness, diagnosis, and comorbidities), and fresh stool samples or rectal swabs will be collected after recruitment. During the intervention and hospital stay, data on clinical progress, investigations (blood, microbiological, radiological), medications and antibiotics, dietary intake, and probiotics/prebiotics use. On day 15, the stool sample or rectal swab will be collected after the investigational product consumption. At discharge/death/referral, the final diagnosis, clinical condition, outcome, medications given, investigation findings, and isolated organisms (any sample source) will be documented. If the patient continues to be hospitalized beyond day 15, an additional stool sample or rectal swab will be collected at the time (within 24 hours) of discharge. During the hospitalization period, a stool sample or rectal swab will be collected for testing if CDI is suspected in any participant (with fever, diarrhea, abdominal pain, or distension). The participants will be followed up (physically/telephonically) on days 30 (+7), 60 (+7), and 90 (+7) post-recruitment to document health status, any illness/infection, medication use, hospitalization, investigations, dietary practices, and probiotic use. The data will be collected using structured case record forms (CRFs).

Adherence and tolerance to the investigational product shall be documented daily during hospitalization. Refusing to participate or withdraw will not affect the participant’s standard care in the hospital. Any untoward medical occurrence, irrespective of the causal relationship, will be recorded and investigated in detail. The serious adverse events (SAEs) shall be evaluated, and causality shall be assigned as unrelated, unlikely, possible, probable, definite, or not assessable.

Outcome Assessment

The CD colonization status will be documented by culture of stool or rectal swabs collected on days 0 and 15. The stool or rectal swab samples will be subjected to glutamate dehydrogenase (GDH) and toxin assay (by enzyme immunoassay, EIA) tests for the participants with suspected CDI. The samples positive for GDH and/or toxin shall be subjected to a nucleic acid amplification test (NAAT). In confirmed cases of CDI, culture for CD (using selective media and anaerobic gas pack) shall be done. The positive culture isolates shall be subjected to VITEK-2 for antimicrobial susceptibility testing (AST). The sample aliquots shall be stored at -80°C for further analysis.

The CD-positive isolates from 30 participants (10 each of those who acquired CD, who eliminated CD, and who had confirmed CDI) shall be subjected to whole genome sequencing. The fecal cytokines (e.g., CXCL-5, CXCL-8) and lactoferrin shall be estimated by enzyme-linked immunosorbent assay (ELISA) kit in a subset of 25 participants per group from stool or rectal swab samples collected on days 0 and 15. The *Torani* product samples will be cultured to detect microbiota (e.g., *Lactobacillus* species), followed by the bacteria species identification (16S rRNA gene amplification) and analysis of SCFAs by high-performance liquid chromatography (HPLC).

Statistical analysis

Sample Size Calculation

A study documented that probiotic consumption reduced CDI by approximately 70% [27]. Assuming that the *Torani*-xylitol mix shall reduce the CD colonization from 15% to 5% in hospitalized participants at discharge, the estimated sample size is 196 participants (98 per group) with a 95% confidence level and 80% power. Thus, we propose to include 200 participants (100 per group).

Data Analysis

The normality of the data distribution will be gauged using the Shapiro-Wilk test. The continuous variables will be expressed as mean and standard deviation or median and interquartile range based on the distribution pattern. The categorical variables will be expressed as frequency and proportion. Missing data will be addressed using various imputation techniques, and sensitivity analyses will be conducted to assess the robustness of the results. The quantitative data with normal distribution will be compared using the unpaired t-test, and those with skewed distribution will be analyzed using the Mann-Whitney U test. The categorical data will be compared using the chi-square or Fisher's exact test, as applicable. Analysis of variance (ANOVA) or the Kruskal-Wallis test shall be considered for the multiple comparisons. A p-value of < 0.05 will be considered significant. For the study outcomes, results will be summarized in absolute (risk difference or mean difference) and relative (risk ratio) effect size measures along with their 95% confidence intervals. Regression analysis shall be done to identify the factors that influence CD colonization or infection and their strength of association. A per-protocol (PP) analysis will be performed to assess efficacy. The safety analysis will be done using the intent-to-treat (ITT) principle. R software (version 4.5.0 or higher) will be used for the data analysis and generation of plots [28].

Interim Analysis

One interim analysis is proposed after enrollment of 50% (n = 100) of participants and completion of the day-15 data. The interim analysis will be conducted using the O’Brien-Fleming spending function and a type I error rate of 5%.

Ethical Consideration and Trial Registration

The Institutional Ethics Committee of KIMS, Bhubaneswar, has approved the study protocol (KIIT/KIMS/IEC/1753/2024). The trial has been prospectively registered with the Clinical Trial Registry of India (CTRI/2025/03/083479). The study will comply with the Good Clinical Practice (GCP), the Declaration of Helsinki, and institutional norms. All the participants will be recruited for the study after obtaining written informed consent, and their privacy and confidentiality will be maintained.

## Results

**Figure 1:**
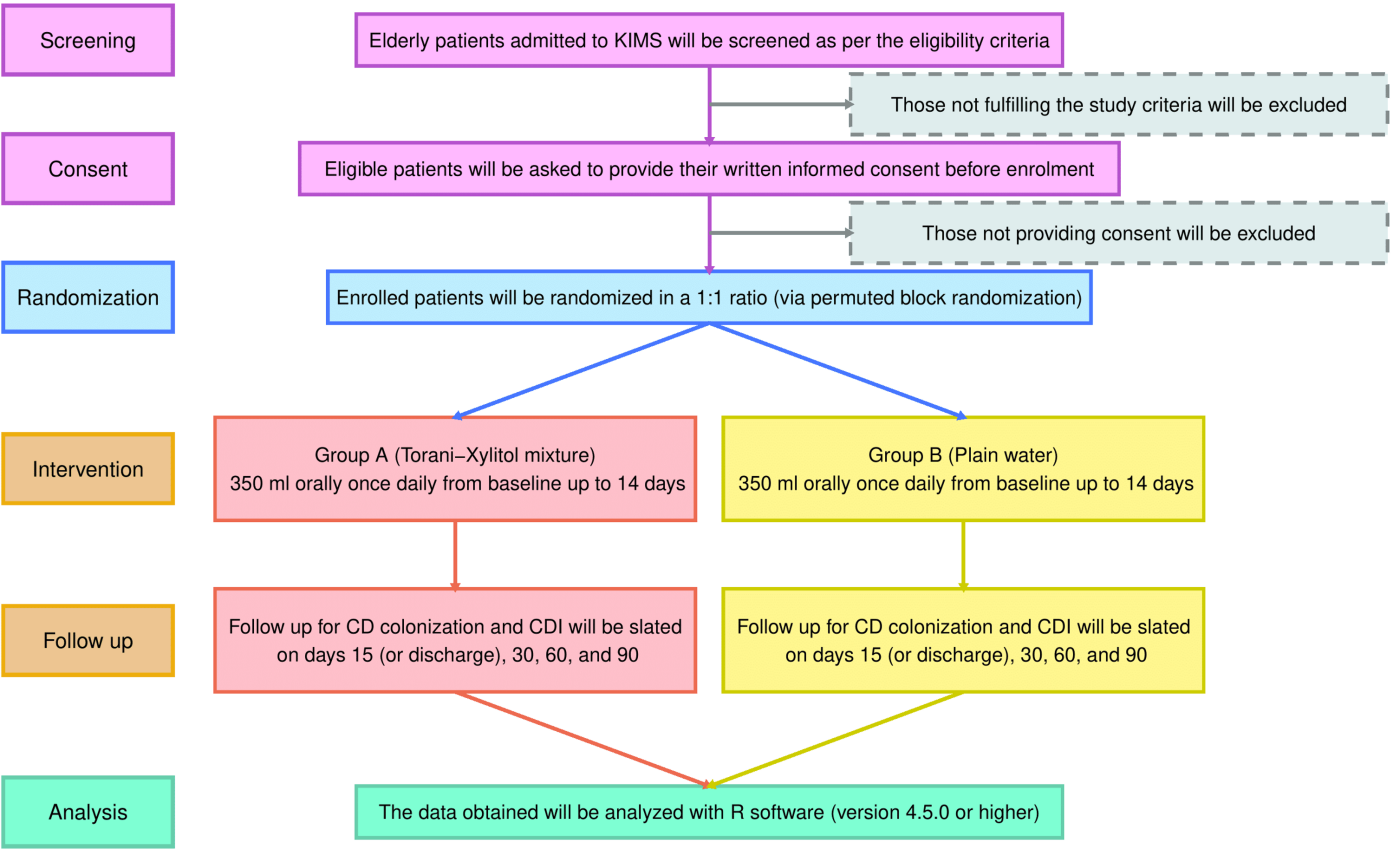
CONSORT diagram CD: *Clostridioides difficile* (also known as *Clostridium difficile*), CDI: CD infection, CONSORT: consolidated standards of reporting trial.

Since the study has not commenced, the results have yet to be obtained. The flow of this RCT has been illustrated in Figure 1.

## Discussion

This RCT has been designed to document the effect of the *Torani*-xylitol mixture on CD colonization and CDI by modulating gut microbiota among hospitalized elderly patients. The outcome measures are CD colonization, CDI incidence, CD profile (genotype, phenotype, and AST) of CD, and safety of the investigational product. As gut dysbiosis is an important causative factor for CDI, the probiotic-prebiotic-synbiotic products have potential roles [11,14]. The traditional probiotic-rich foods like *Torani* (fermented rice water) in Odisha provide advantages in their acceptability, ease of preparation, availability and use, and affordability. The components of *Torani*, like SCFA and enrichment with prebiotic (xylitol), are likely to repair gut dysbiosis, modulate colonic milieu, improve gut immunity, and prevent CD colonization and CDI [14,15,17,19].

This study has a few limitations. First, the study design is an open-label RCT, owing to the nature of the intervention. Allocation concealment will be done to mitigate the performance and detection bias. Second, the duration of therapy is fixed for all participants irrespective of the group, gender, disease, or hospitalization duration. Third, we could not get the exact power analysis for this study, as this is the first study to document the effect of the *Torani*-xylitol mixture on CD colonization and CDI.

## Conclusions

To the best of our knowledge, this is the first RCT to document the effect of the *Torani*-xylitol mixture on CD colonization and CDI in at-risk elderly hospitalized patients. We hope the combination of probiotics and prebiotics assists gut dysbiosis repair, reducing CD colonization and CDI. If the findings support the hypothesis, it will aid in promoting traditional fermented food usage for preventive, promotive, and therapeutic purposes to reduce morbidity, mortality, hospitalization rate and duration, and cost.
